# Loss of Calponin 2 causes premature ovarian insufficiency in mice

**DOI:** 10.1186/s13048-024-01346-y

**Published:** 2024-02-09

**Authors:** Tzu-Bou Hsieh, Jian-Ping Jin

**Affiliations:** 1 Department of Obstetrics & Gynecology, Wayne State University School of Medicine, Detroit, MI 48201 USA; 2 Department of Physiology, Wayne State University School of Medicine, Detroit, MI 48201 USA; 3 Department of Physiology and Biophysics, University of Illinois at Chicago College of Medicine, Chicago, IL 60612 USA

**Keywords:** Calponin 2, Cytoskeleton, Granulosa cells, Premature ovarian insufficiency

## Abstract

**Background:**

Premature ovarian insufficiency (POI) is a condition defined as women developing menopause before 40 years old. These patients display low ovarian reserve at young age and difficulties to conceive even with assisted reproductive technology. The pathogenesis of ovarian insufficiency is not fully understood. Genetic factors may underlie most of the cases. Actin cytoskeleton plays a pivotal role in ovarian folliculogenesis. Calponin 2 encoded by the *Cnn2* gene is an actin associated protein that regulates motility and mechanical signaling related cellular functions.

**Results:**

The present study compared breeding of age-matched calponin 2 knockout (*Cnn2*-KO) and wild type (WT) mice and found that *Cnn2-*KO mothers had significantly smaller litter sizes. Ovaries from 4 weeks old *Cnn2-*KO mice showed significantly lower numbers of total ovarian follicles than WT control with the presence of multi-oocyte follicles. *Cnn2-*KO mice also showed age-progressive earlier depletion of ovarian follicles. *Cnn2* expression is detected in the cumulus cells of the ovarian follicles of WT mice and colocalizes with actin stress fiber, tropomyosin and myosin II in primary cultures of cumulus cells.

**Conclusions:**

The findings demonstrate that the loss of calponin 2 impairs ovarian folliculogenesis with premature depletion of ovarian follicles. The role of calponin 2 in ovarian granulosa cells suggests a molecular target for further investigations on the pathogenesis of POI and for therapeutic development.

## Background

Reproductive ageing in women is signified by the accelerated declining in ovarian follicle number and poor egg quality. This natural process occurs progressively and leads to total loss of fecundity, cycle regularity, and eventually menopause ensued at around age 50. Premature ovarian insufficiency (POI) is defined as menopause occurred before 40 years of age. Patients with POI exhibit a sharp declining in fecundity a decade before the onset of menopause. There is no effective treatment for patients with POI while early diagnosis and identification of patients impending POI or poor ovarian reserve for intensive treatment with assisted reproduction technology may achieve limited improvement in pregnancy outcomes [1]. The early cessation of ovarian function also causes other medical conditions such as osteoporosis, skin changes, cardiovascular diseases and memory impairment [2]. POI can result from various diseases, surgery, radiation and chemical toxicity or be idiopathic. The exact mechanisms causing primary premature ovarian failure remain unknown [3].

Ovarian reserve established during fetal development is the major factor determining the longevity of ovarian function. Ovarian folliculogenesis is a long process starting from embryonic stage and throughout the entire reproductive period. Primordial germ cells (PGCs) were the first identifiable germ cells with rapid proliferation potential originated from the yolk sac. After migrating to the genital ridge, PGCs continue to proliferate until E13.5 in mice when a portion of them stops mitotic division and develops into oogonia during sexual differentiation to later form oocytes. After entering meiosis, the oocytes are arrested at the diplotene stage of prophase of meiosis I until puberty [4, 5]. More than half of the oocytes are lost by apoptosis and the remaining oocytes are assembled to form primordial follicles, while a significant number of follicles degenerates throughout the reproductive life [6, 7].

Ovarian reserve is the total number of dormant ovarian follicles that can be used for reproduction and determines the total number of ovulatory cycles. A balance between germ cell mitosis and primordial follicle apoptosis determines the volume of the ovarian reserve. In mice, the number of ovarian follicles reaches a peak before birth and constitutes the ovarian reserve. After birth, the ovarian follicles are mainly composed of primordial or primary follicles, both of which are unresponsive to gonadotropin stimulation. In 3–4 weeks old mice, some of the primordial follicles are activated with the proliferation of the granulosa cells and enlargement of the oocyte to form the secondary, or antral follicles. As all ovarian follicles are under the same gonadotropin stimulation profile, the exact mechanisms determining a specific number of the primordial follicles being recruited to enter each wave of the ovulatory cycles while the rest of the follicles remain quiescent are not fully understood. Besides intraovarian paracrine or humoral factors that may function in this regulation, recent studies have shown that mechanical tension plays a role in controlling the selection and maintaining dormant state of the primordial follicle in mice [8, 9].

In vertebrate, the outer surface of the ovary is covered by a single layer of the germinal epithelium and the underlying tunica albuginea made of dense collagen fiber. The tunica albuginea supports the ovarian follicles and connective tissue in place and maintain a constantly fluctuating intraovarian pressure. At the initiation of an ovulation cycle, a wave of the ovarian follicles is activated and enlarge in size with rapid proliferation of the granulosa cells in response to the stimulation of gonadotropins as well as accumulation of the follicular fluid until ovulation occurs. In human, the size of the growing follicles increases from the primary follicles of less than 0.1 mm diameter to almost 20 to 27 mm average diameter of the Graafian follicles [10] generates an increasing intraovarian pressure before ovulation. During this period, a more than 10 folds increase in the volume of the growing follicles exerts high pressure to the adjacent small follicles and inhibit their development. A previous study demonstrated that the minimal developmental time needed for primary follicles to reach the antral stage is approximately 12 days [11], which is much longer than the duration of around 8 days in in vitro culture of isolated ovarian follicles. This observation suggests that intraovarian tension inhibits follicular development and the actin cytoskeleton may play a role in mediating the inhibitory effect of mechanical tension on ovarian follicles.

Calponin is an actin microfilament associated protein that regulates cytoskeleton functions via inhibiting actin-activated myosin ATPase and motor activity [12–14]. Three isoforms of calponin, calponin 1, calponin 2 and calponin 3, have evolved in vertebrates encoded by homologous genes, *Cnn1, Cnn2 and Cnn3*, respectively [15, 16]. Calponin 1 is exclusively expressed in differentiated smooth muscle cells and is the most studied isoform for its function in the regulation of smooth muscle contractility [15, 17, 18]. Calponin 3 is expressed in smooth muscle cells [19], brain [20], trophoblasts [21] and lymphocytes [22, 23]. Calponin 2 is expressed in smooth muscles [24] and in several types of non-muscle cells, such as fibroblasts [25], epithelial cells [26], endothelial cells [27] and macrophages [28]. Calponin 2 regulates actin-cytoskeleton-based cellular functions, such as migration [29] and cytokinesis [30] through modulating the tension and stability of actin filaments [31] via inhibitory regulation of myosin II dependent cell traction force [32]. The gene expression and protein stability of calponin 2 are both regulated by mechanical tension [25]. It has been demonstrated that the average cytoskeleton tension over time, other than dynamic tension, determines the level of calponin 2 expression [26].

Previous studies have detected calponin 2 expression in the ovary [26] and it regulates cell proliferation [24, 30], suggesting that calponin 2 may play a role in ovarian folliculogenesis. In the present study, we investigated the impact of *Cnn2* gene deletion on ovarian functions. Studies of *Cnn2-*KO mice and primary culture of ovarian cumulus cells demonstrated that calponin 2 is expressed in the cumulus cells and the loss of calponin 2 impaired ovarian folliculogenesis with premature depletion.

## Results

### *Cnn2*-KO and wild type (WT) mice show no significant difference in overall development and life expectanc**y**

**Fig. 1:**
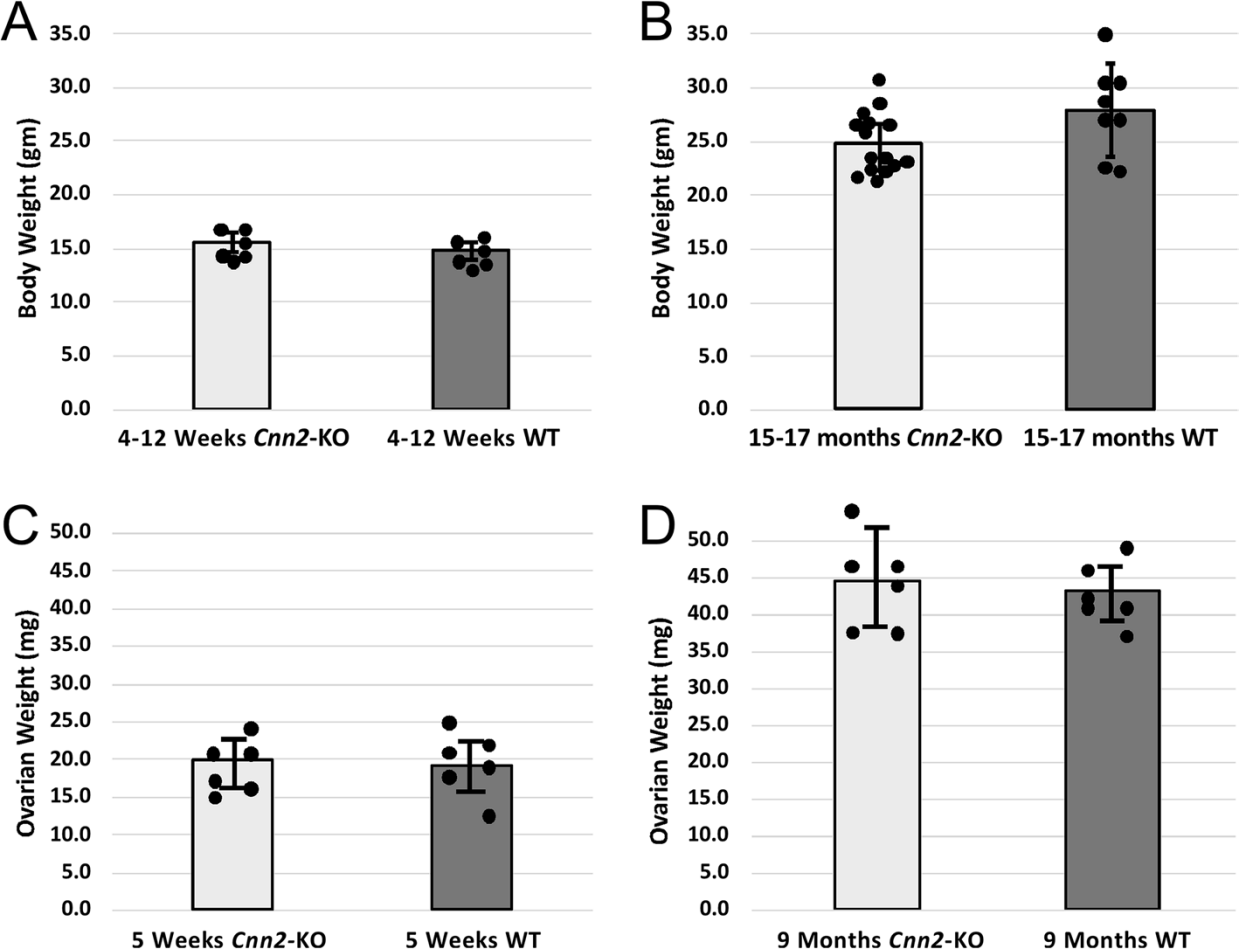
No significant differences in body weight and ovarian weight between *Cnn2*-KO and WT mice. **A** The body weight of *Cnn2*-KO mice at 4 to12 weeks old was 15.3 ± 0.83 gm, similar to that of WT control (14.6 ± 0.86 gm). *p* = 0.19. **B** The body weight of *Cnn2*-KO mice at 15 to 17 months old was 25.0 ± 2.71 gm, also not statistically different from WT control (27.9 ± 4.30 gm) *p* = 0.06. **C** The ovarian weight of *Cnn2*-KO mice at 5 weeks old was 19.9 ± 3.93 mg, similar to that of WT control (19.3 ± 3.86 mg), *p* = 0.79. **D** The ovarian weight of *Cnn2*-KO mice at 9 months old was 44.6 ± 6.55 gm, also similar to the WT control (43.1 ± 3.72 gm), *p* = 0.63. Data are shown as mean ± SD, Statistical comparison was done using paired Student *t* test

Genotypes of all mice studied were determined on DNA extracted from tissue biopsies using polymerase chain reaction (PCR) and confirmed after experimental study using Western blot analysis of post-mortem protein samples from the spleen (data not shown). Consistent with our previously published results [28, 33–35], homozygous *Cnn2-*KO mice show normal life expectancy in non-stressed cage environment and could live up to 28 months old. The statistically analyzed quantitative data in Fig. 1 show no significant differences in body weight and ovarian weight between WT and homozygous *Cnn2-*KO mice at young and older ages. The ovarian weight of *Cnn2*-KO mice at 5 weeks old was 19.9 ± 3.93 mg similar to that of WT mice (19.3 ± 3.86 mg). The ovarian weight of *Cnn2*-KO mice at 9 months old was 44.6 ± 6.55 gm, also similar to the WT control (43.1 ± 3.72 gm).

### Homozygous *Cnn2*-KO female mice are fertile but produce litters of significantly smaller size than WT controls

Both homozygous and heterozygous *Cnn2-*KO mice produce offspring naturally. The offspring genotypes summarized in Table 1 show a striking finding that homozygous female *Cnn2*-KO mice produced significantly smaller litters than that of WT females. The mating with either *Cnn2*-KO or WT males made no significant difference, indicating a female-specific low reproduction performance due to the deletion of calponin 2. Male homozygous *Cnn2*-KO mice paired with WT female mice produced normal litter sizes, confirming that *Cnn2*-KO does not affect male reproductivity.

**Table 1:**
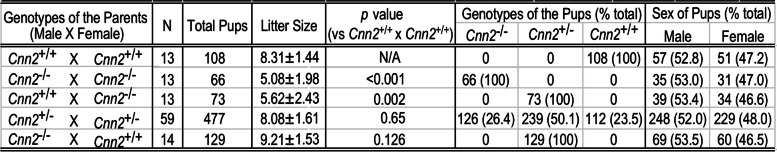
Female Cnn2-KO mice produced significantly smaller litter size than control groups

The litter size from *Cnn2* ^+/−^ heterozygous parents was similar to WT control with normal Mendelian segregation (Table 1). The results indicate that homozygous or heterozygous *Cnn2*-KO fetus has developmental potential comparable to WT fetus. No sex bias was seen among the offspring from homozygous, heterozygous and WT parents. The data from breeding with *Cnn2* ^−/−^ and *Cnn2* ^+/−^ males (Table 1) demonstrate that X and Y *Cnn2*-KO sperms are both normally developed and retain their ability of fertilization.

### Homozygous female *Cnn2*-KO mice show premature age-progressive declining of litter size

**Fig. 2:**
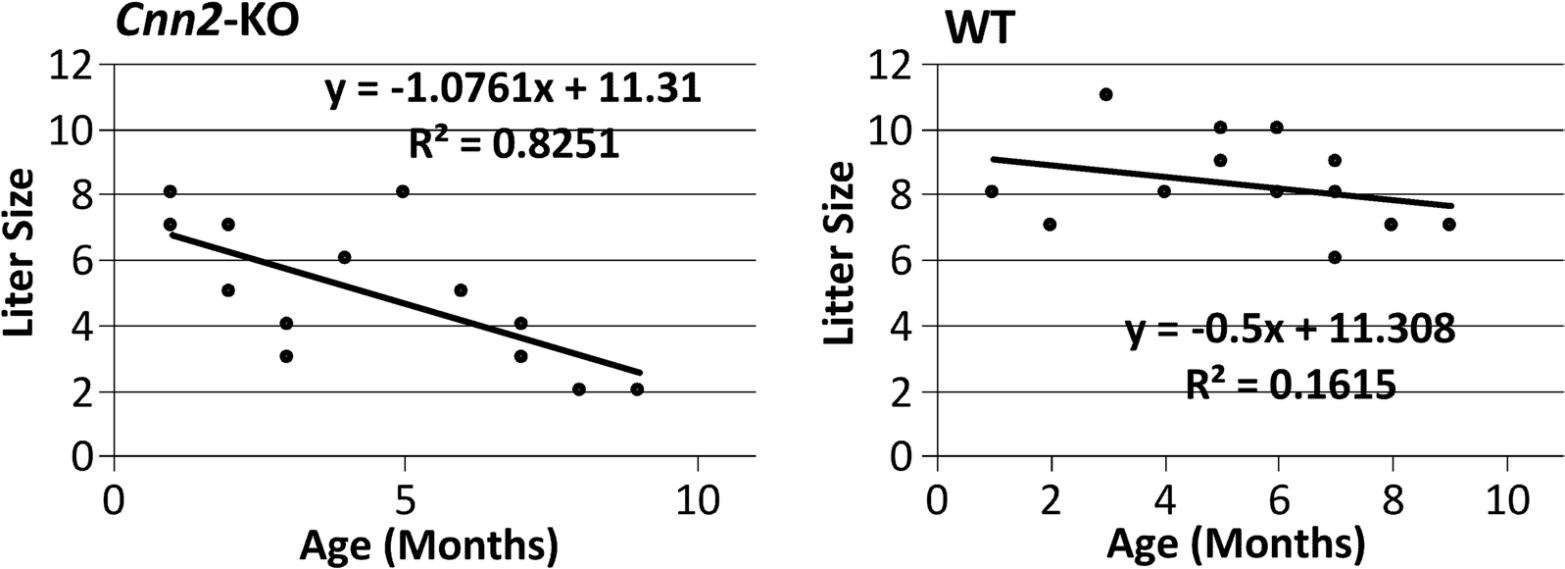
*Cnn2* ^−/−^ female mice exhibited age-progressive decline in reproductive litter size. The scattered plots demonstrate a significant negative correlation of the litter size with maternal age of *Cnn2-*KO female breeders (from mating at 1–9 months old) (*p* = 0.006 two-tail Pearson’s correlation analysis). The litter size of age-matched WT mothers remained stable during this age period (*p* = 0.349)

The scattered plots in Fig. 2 show the trends of litter size produced from one- to nine-months old female *Cnn2-*KO and WT mice. The results demonstrate that homozygous *Cnn2-*KO mice exhibit a premature age-progressive declining in litter sizes while the WT controls remained stable during this time period. Homozygous *Cnn2*-KO female mice produced offspring at the same initial age of reproduction (one month old) as that of WT female mice, demonstrating that the deletion of calponin 2 does not affect the onset of puberty in mice. The similar litter sizes of one month old *Cnn2-*KO and WT mice also indicate that *Cnn2*-KO did not impair the systemic capacity of carrying normal number of embryos at young age.

### Young *Cnn2*-KO mice show significantly lower total ovarian follicle counts and high numbers of atretic follicles

**Fig. 3:**
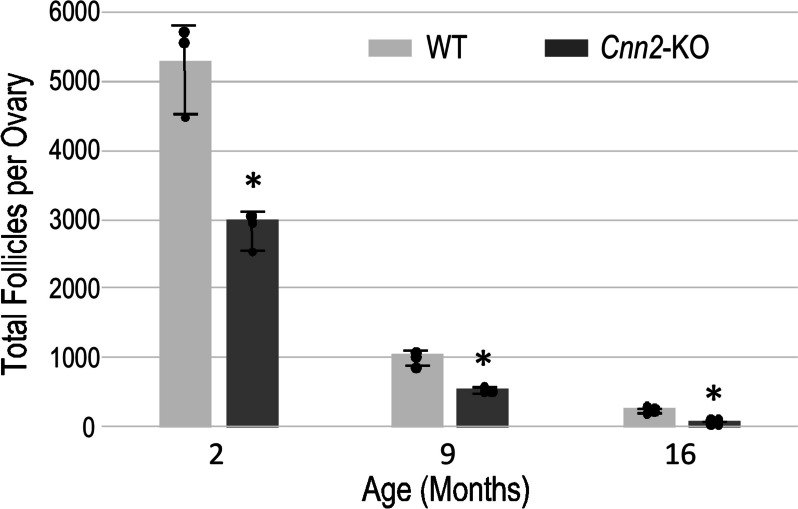
*Cnn2*-KO mice show low total ovarian follicle counts at young age and age progressive premature depletion. The data demonstrate that *Cnn2-*KO mice had drastically lower total ovarian follicle counts than WT control at 2 months old. The difference became larger in 9 and 16 months old groups. *N* = 3 mice each for the 2 and 9 months old groups and 4 for the 16 months old group. *p < 0.05 in Student t test

Serial sections of whole ovaries from three pairs each of young and older homozygous *Cnn2-*KO and WT mice were examined histologically and compared for ovarian follicle counts. The results in Fig. 3 and Table 2 show that homozygous *Cnn2-*KO mice had significantly lower total ovarian follicle counts than WT controls at 2, 9 and 16 months old. The total ovarian follicle counts of 2-month-old *Cnn2-*KO mice are less than 60% of the counts of age-matched WT control, indicating impaired ovarian folliculogenesis and low ovarian reserve. The difference between *Cnn2-*KO and WT groups became larger at 9 and 16 months of age (Fig. 3), demonstrating that *Cnn2*-KO causes faster depletion of ovarian follicles.

**Table 2:**
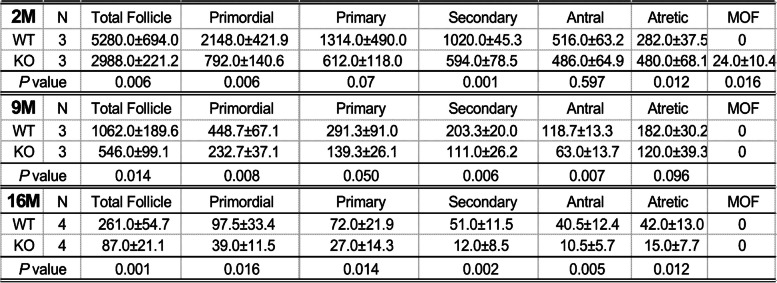
Comparison of ovarian follicle counts in young and aging WT and *Cnn2*-KO mice

We further categorized and compared different stage of the ovarian follicle count at different ages (Table 2). The average number of the primordial follicle in *Cnn2*-KO mice showed significant decreases from 2 months to 9 and 16 months of age. Interestingly, antral follicle counts of 2 months old *Cnn2*-KO mice had no significant difference from the WT control whereas they showed a significant decrease in *Cnn2*-KO mice at 9 and 16 months old as compared to WT controls. These findings demonstrate that *Cnn2*-KO mice have comparable ovulatory function at young age, which was significantly declined in older animals, consistent with the reproductive outcome results.

**Fig. 4:**
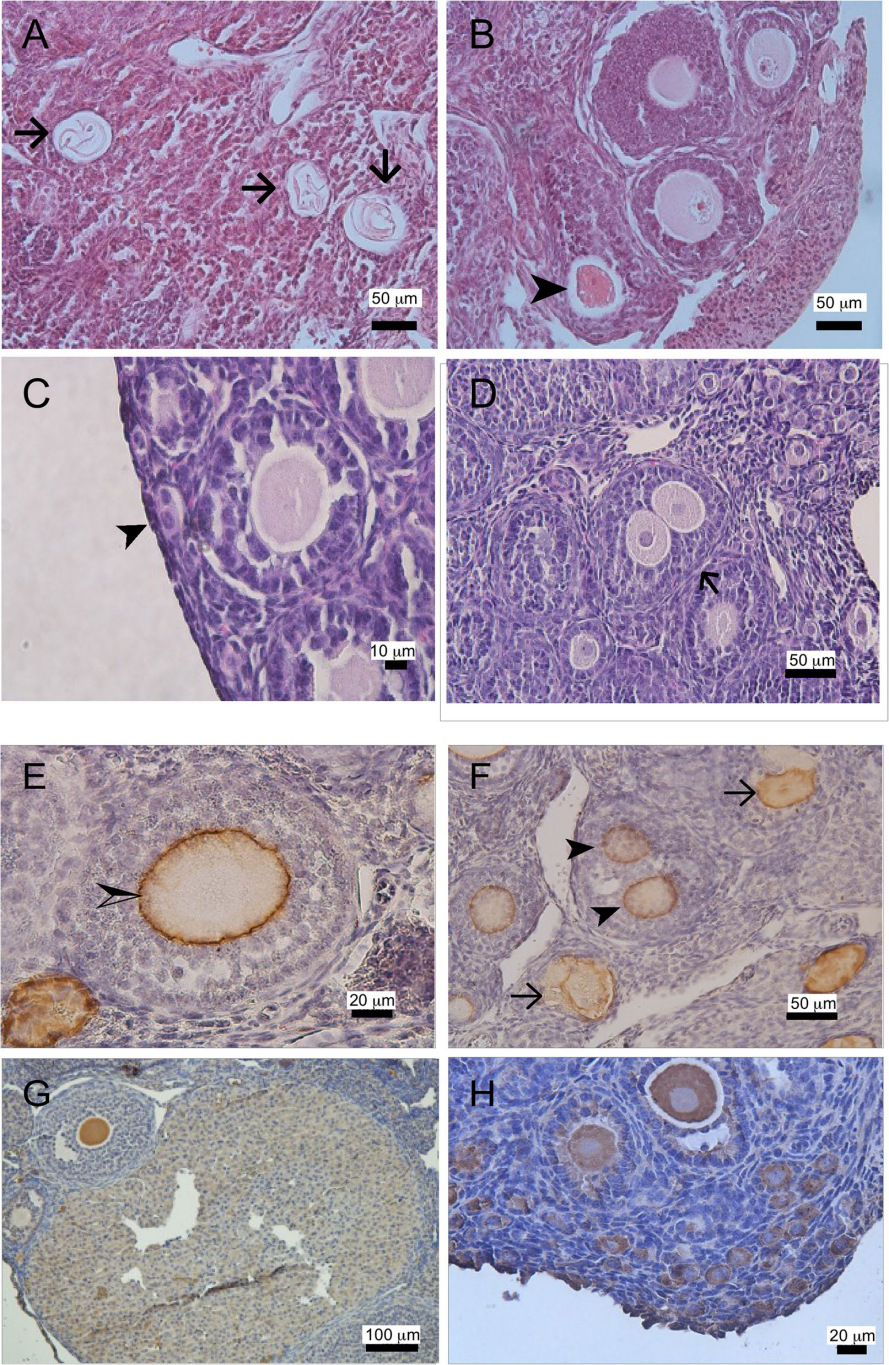
Morphology of atretic follicles and MOFs. **A** An example of ovarian section from an 8 weeks old homozygous *Cnn2*-KO mouse shows atretic follicles containing collapsed oocytes with only empty zona pellucida remaining (→). **B** An example of a WT mouse ovarian section shows atretic follicles containing dark red-stained oocytes (➤). **C** & **D** The example sections show multi-oocyte follicles at different stage of development, from primordial follicle (➤) and secondary follicles (→). A and B are H&E staining, and C and D are PAS staining images. **E** ZP-3 expression in the zona pellucida of a normal preantral follicle (half arrow) of WT mouse. **F** ZP3 expression in zona pellucida of a multi-oocyte follicle (arrowhead) and degenerated follicles (arrow) of *Cnn2*-KO mouse. The oocytes with irregular shaped or collapsed zona pellucida indicate oocyte degenerations. **G** FSHR was expressed in the granulosa cells and oocytes of antral follicles as well as in the corpus luteum of *Cnn2*-KO mouse. **H** VASA was expressed in oocytes in primordial, primary, secondary and antral follicles of *Cnn2*-KO mouse

Comparing to WT control, the ovaries of young *Cnn2-*KO mice also had higher numbers of atretic follicles. Figure 4A-D show representative images of the ovarian histology from an 8 weeks old homozygous *Cnn2-*KO mouse. Figure 4E-H verified the expression of ZP3 in zona pellucida of normal follicles and multi-oocyte follicles (MOFs), and the expression of DEAD box polypeptide 4 (VASA) as well as follicle stimulating hormone receptor (FSHR) in ovarian follicles. Most of the atretic follicles displayed collapsed oocytes with only empty zona pellucida remained. Abnormal follicles such as MOFs were present in the ovaries of young *Cnn2-*KO mice at various stages of folliculogenesis, ranging from primordial follicles, primary follicles to secondary follicles. In contrast, the atretic follicles in the ovaries of WT mice showed oocytes stained dark red in H&E staining with no MOF present.

### *Cnn2*-KO mice exhibit early depletion of ovarian follicles

**Fig. 5:**
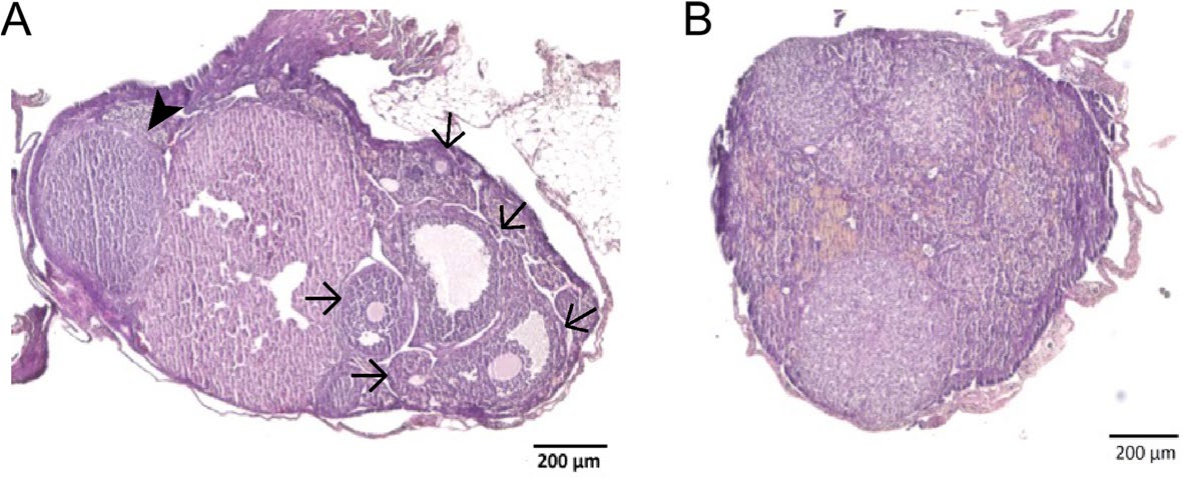
*Cnn2*-KO mice show complete depletion of growing follicles at 16 months of age. **A **Representative histological image of a 16 months old WT mouse ovary showed notable ovarian follicles (arrows: ovarian follicles, arrowhead: corpus luteum) whereas 16 months old *Cnn2-*KO mouse ovary showed complete loss of the growing follicle (**B**). It is interesting that the ovary of 16 months old *Cnn2-*KO mouse shows many remnants of the corpus luteum but without any observable growing follicle. While the present study focuses on the depletion of ovarian follicles, the effect of *Cnn2-*KO on generating the corpus luteum is worth investigating in future studies

Table 2 shows that while 16 months old WT mouse ovaries still contain hundreds of ovarian follicles, the ovaries of *Cnn2*-KO mice at this age had very low numbers of primordial follicles and no growing follicles were detected. Figure 5A shows examples of remaining ovarian follicles in the ovary of a 16 months old WT mouse. The ovarian histology of a 16 months old *Cnn2-*KO mouse in Fig. 5B showed that the ovary was filled with fibrotic tissue and degenerated extracellular matrix with no growing follicle of any stage. Our previous studies demonstrated that deletion of calponin 2 attenuates inflammatory responses and fibrosis [33, 36]. Therefore, it is likely that fibrosis and extracellular matrix degeneration are consequences rather than causes of ovarian follicle depletion in *Cnn2*-KO mice.

### Calponin 2 is expressed in oocytes and cumulus cells

**Fig. 6:**
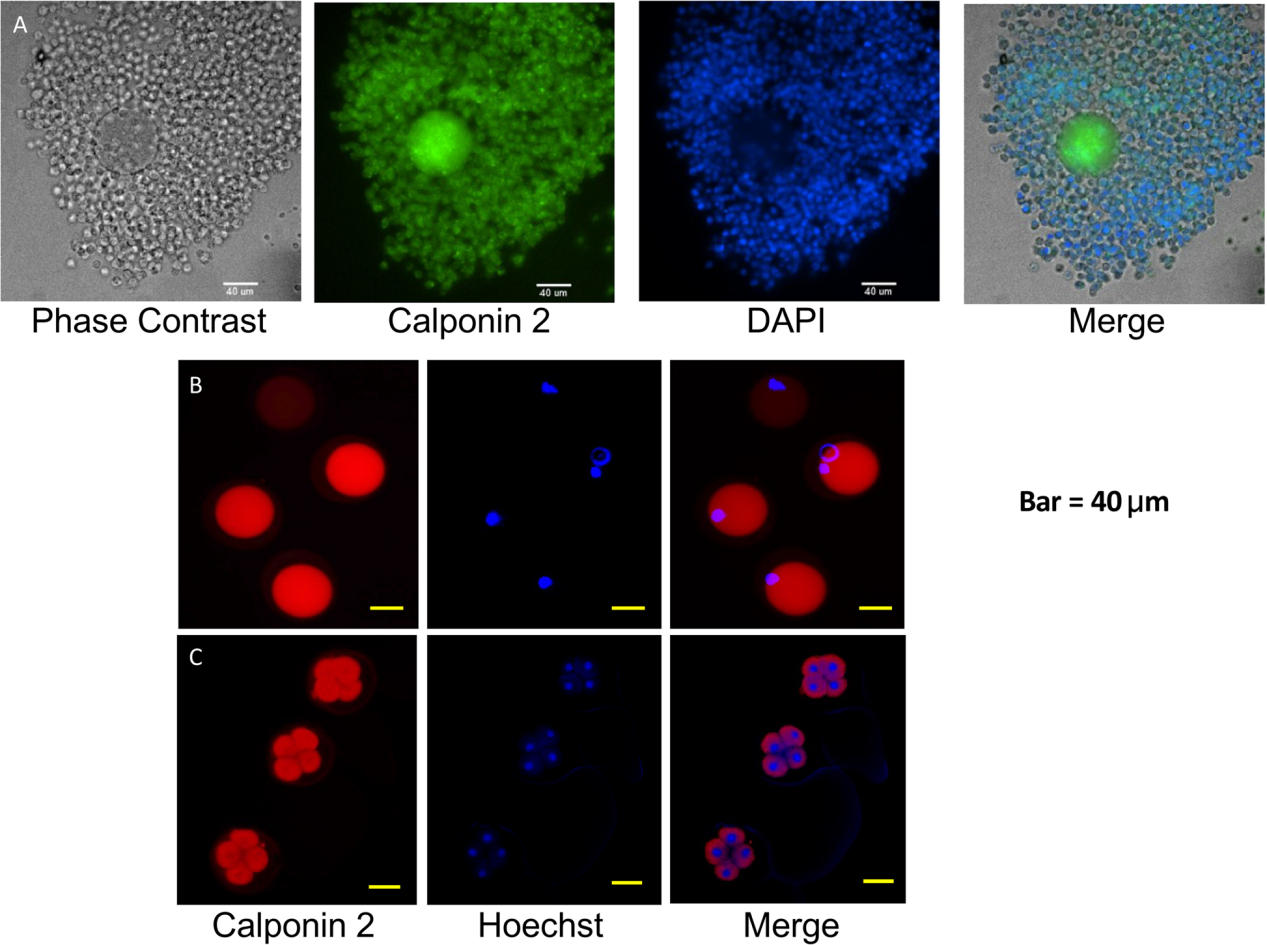
Calponin 2 expression in oocytes and cumulus cells. **A** Cumulus-oocyte-complexes (COCs) were collected from oviducts of a young WT female mouse 12 h after stimulated superovulation. After hyaluronidase digestion and removal of the cumulus cells, the COCs was stained with anti-calponin 2 mAb 1D11 and DAPI or Hoechst for nuclei. The representative images of a COC show that calponin 2 is expressed in both the oocyte and the surrounding cumulus cells. **B** Calponin 2 is detected homogenously in the ooplasm of the oocyte. **C** mAb 1D11 staining of a 4-cell embryo showed calponin 2 expression in the peripheral of the blastomeres. Bar = 40 μm

Figure 6A shows immunofluorescent microscopic image of a cumulus-oocyte-complex (COC) stained with anti-calponin 2 mAb 1D11 [26] and DAPI for nuclei. The representative image shows that calponin 2 was present in the cytoplasm of oocyte and the surrounding cumulus cells. Figure 6B demonstrates mAb 1D11 and Hoechst stains in isolated oocytes after hyaluronidase digestion and removal of the cumulus cells, in which calponin 2 was detected homogeneously in the cytoplasm. Figure 6C shows three 4-cell stage embryos collected from a female WT mouse after timed matting. The embryos were stained with anti-calponin 2 mAb 1D11 and Hoechst for nuclei of cells, in which calponin 2 expression was detected at the peripheral of the blastomeres.

### Calponin 2 is expressed in cultured granulosa cells

**Fig. 7:**
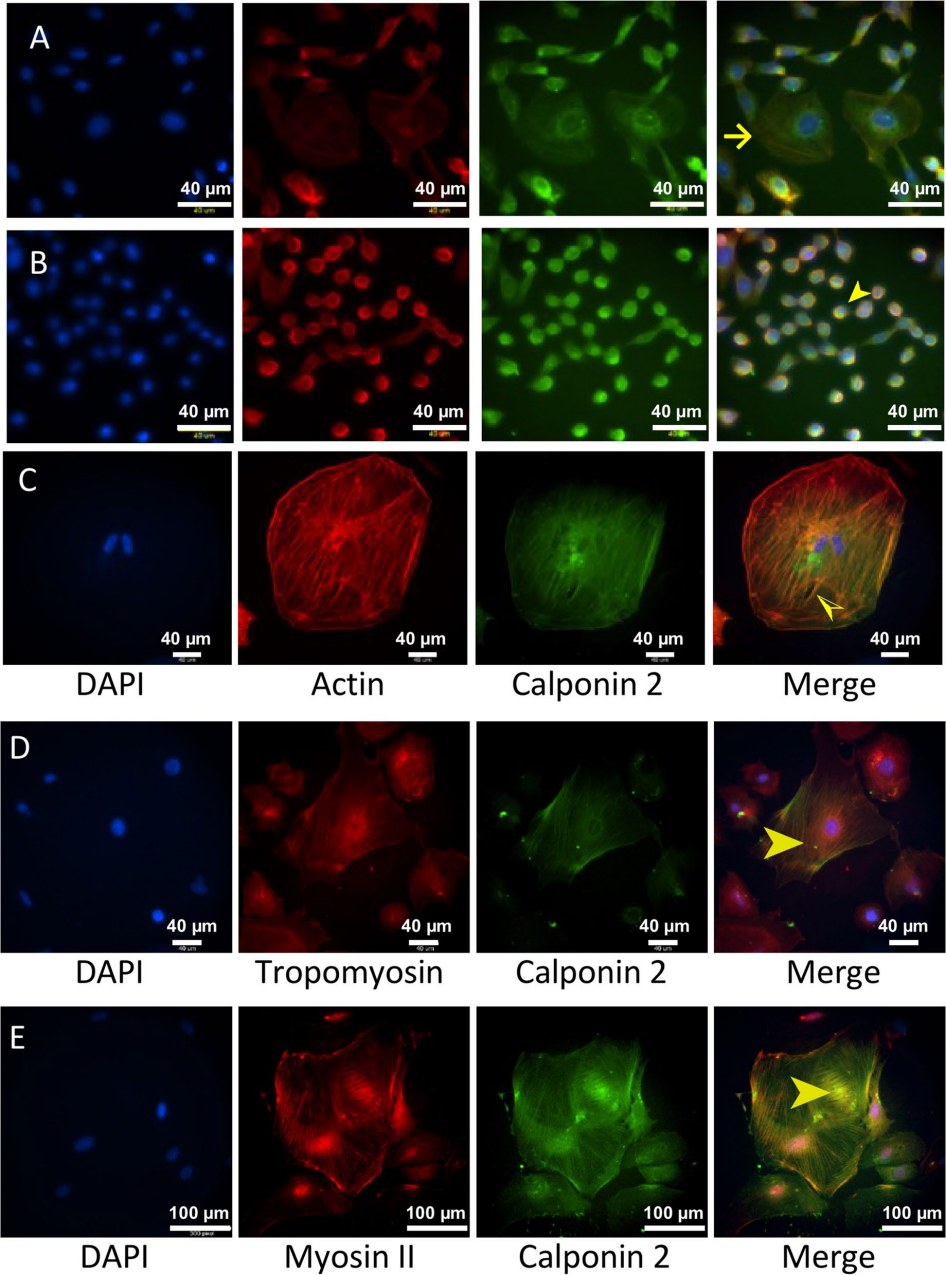
Calponin 2 colocalizes with actin microfilaments, tropomyosin, and myosin II in ovarian granulosa cells. Granulosa cells were isolated from young female WT mice and examined with immunofluorescence microscopy after culturing for 48 h. **A** The representative images show that the granulosa cells are of polygonal epithelial-like morphology with flat and wide spreading cytoplasm (the arrow). **B** The arrowhead indicates a group of the granulosa cells displaying a cobble stone like appearance. **C** The cultured granulosa cells show significantly variable spreading areas of which some are much larger than others. The image of an example of large cells shows actin stress fibers in the cell body and on the edge of the cells and the colocalization of calponin 2 (the half arrowhead). **D** Colocalization of calponin 2 with tropomyosin (the arrowhead). **E** Colocalization of calponin 2 with myosin II (the arrowhead). F-actin was stained with rhodamine phalloidin. Nucleus counterstain was using DAPI

After isolation and cultured for 48 h, WT mouse ovarian granulosa cells showed different sizes and various morphologies. One group of the granulosa cells in low density culture exhibit epithelial-like morphology (Fig. 7A) with the cells widely spreading in hexagonal or spindle shapes. Another group of the granulosa cells display a cobble stone like appearance (Fig. 7B) when the cells reached higher confluency. Actin stress fibers are seen as filamentous threads stretched out in the cytoplasm of primary cultures of ovarian granulosa cells. Calponin 2 in ovarian granulosa cells is colocalized with actin microfilaments (Fig. 7C), tropomyosin (Fig. 7D) and myosin II (Fig. 7E), implicating a role in regulating cytoskeleton structure and tension-related functions.

## Discussions

The present study demonstrated for the first time that calponin 2 plays a critical role in ovarian development and aging. To explore the role of *Cnn2* in female fertility and in the pathogenesis of POI, our novel findings lead to the following observations.

### *Cnn2*-KO mice provide a novel animal model to study POI

The spectrum of idiopathic POI is profoundly variable. The earliest symptoms of POI can be asymptomatic or be noted by menstrual irregularity or anovulation and may take years to develop hypoestrogenism or menopausal symptoms [37]. To date, there is no ideal animal model for studying the disease process of idiopathic POI. The reproductive phenotypes of homozygous *Cnn2* deletion in mice mirror the clinical scenario of idiopathic POI and poor ovarian reserve in humans. Most of the current transgenic mouse models for POI involve mutations that result in complete sterilization or loss of primordial germ cells at neonatal or early prenatal period [38–41], imposing limitations to studying the natural course of idiopathic POI. Mice treated with anticancer drugs are alternatives of animal models for studying POI. Although these models are suitable for research focusing on gonadotoxin-induced POI, they are quite different from the clinical scenarios of idiopathic POI [42].

With fertility and a life expectancy similar to WT mice, *Cnn2-*KO mice provide a new platform for longitudinal investigations to study the progression of POI and a new model system to study the role of ovarian granulosa cells in the pathogenesis of idiopathic POI. In addition, homozygous systemic *Cnn2*-KO mice show no apparent change in overall development and the age of first pregnancy (4 weeks), indicating that deletion of calponin 2 did not change the pubertal development of female mice. Furthermore, *Cnn*2-KO mice have the same life expectancy without apprarent structural or functional defects. Therefore, although a significant number of the calponin 2-null oocytes end up with atresia, fully matured *Cnn2*-KO oocytes still retain their fertilization and development potential. The data that young female *Cnn2*-KO mice generated normal number of pups further indicated unaffected estrus cycles. These features allow us to focus on evaluating the effects of loss of *Cnn2* on ovarian granulosa cell biology and function. In future studies, granulosa cell-specific *Cnn2*-KO mice model could provide information to verify the role of calponin 2 in granulosa cell functions.

### Loss of calponin 2 causes low ovarian reserve and early depletion of ovarian follicles

Total ovarian follicle count is the most important factor determining the life expectancy of the ovarian reserve. Various methodologies have been used to estimate total ovarian follicle counts and the variation of the count could be as high as 10 folds [43]. Although labor-intensive, the method that we employed (see description below) produced consistent ovarian follicle counts comparable to WT mouse data in the literature. Our results demonstrate that female homozygous *Cnn2*-KO mice exhibit low ovarian reserve at young age with progressive premature depletion of ovarian follicles.

*Cnn2-*KO mice also displayed significantly lower numbers of single layered follicles that constitute the major ovarian follicle pool established in the prenatal period. This finding further indicating that the poor ovarian reserve may be due to inadequate primordial follicle formation and/or excessive ovarian follicle apoptosis in the prenatal stage. We also found that *Cnn2-*KO mice have significantly lower numbers of multi-layered follicles which represent the ovarian follicles recruited and activated for further development. This trait could be from accelerated oocyte death or granulosa cell dysfunctions and is worth further investigating.

### Calponin 2 is expressed in oocytes and granulosa cells

Calponin 2 is previously known to express in multiple cell types such as skin keratinocytes, fibroblasts, epithelial cells, endothelial cells and macrophages [16]. Our present study showed that calponin 2 is expressed in oocytes, granulosa cells and in the cleavage stage of early embryos. Since female *Cnn2*-KO mice are fertile with no significant decrease in litter size at 4–8 weeks old (Fig. 2), the loss of calponin 2 in oocytes is unlikely a primary cause of ovarian follicle atresia. The atretic follicles are mostly found in secondary follicles with few primordial follicles. In secondary or antral follicles, oocytes are arrested in the first meiotic division and nutrients need to be supplied by the granulosa cells through the actin-rich transzonal projections. Therefore, loss of calponin 2 function in granulosa cells may be the major cause of follicular atresia.

Immunofluorescence microscopy showed colocalization of calponin 2 and actin stress fibers and myosin II motors in the granulosa cells. An inhibitor of myosin ATPase, calponin 2 in ovarian granulosa cells may function in balancing the myosin motor-based cellular force [28] to regulate cytoskeleton dynamics and connection with neighboring cells. Our previous studies have established the role of calponin 2 in cell migration and cytokinesis. Ovarian granulosa cells are rapidly proliferating from a single layer into multiple layers of the antral follicles. Therefore, impaired cell migration and proliferation may be an underlying mechanism for *Cnn2*-KO to cause POI. Our findings in the present study call for future investigations on ovarian calponin 2 expression and function during early folliculogenesis to fully understand the underlying mechanisms.

### Deletion of calponin 2 causes the formation of multi-oocyte follicles (MOFs)

An intriguing finding in our study was the loss of calponin 2 to result in the generation of MOFs (Table 2). The exact mechanism causing MOFs is unknown. Studies have shown that MOFs could be caused in rodents by abnormal development or migration of granulosa cells during early stage of folliculogenesis [44–46]. The role of calponin 2 in cell proliferation and cell migration may contribute to the formation of MOFs in *Cnn2*-KO mice. MOFs have been proposed to result from abnormal granulosa cell migration, dysregulated follicle assembly, incomplete ovigerous cord breakdown and/or abnormal PGC nest breakdown [47]. Although higher than expected MOFs develop temporarily in peripubertal rats, the existence of MOFs can be used as a marker of abnormal folliculogenesis [46].

Several transgenic animal models have shown increased MOFs, such as mice with a knockout of the BMP15/GDF-9 [48], Notch-2 [49] or FSH/inhibin [50] gene. Previous animal studies showed that fetal susceptibility to environmental toxins, such as bisphenol A, and postnatal exposure to genistein [51], or radiation [52] leads to the formation of MOFs. Our findings in *Cnn2*-KO mice support the early formation of MOFs starting from the primordial follicle stage after nest breakdown. Little is known about the development of MOFs before primordial stage. The effect of calponin 2 deletion on granulosa cell migration or assembly with oocyte to promote the formation of MOFs opens a new area of future investigation for the mechanisms of fetal and perinatal ovarian folliculogenesis and the role of actin cytoskeleton in the fate of PGC nests.

### Calponin 2 as a novel molecular target regulating intra-ovarian mechanical tension signaling in folliculogenesis and POI

Most of the calponin 2 expressing cells reside in a tissue environment of high mechanical tension. Cyclically developed growing ovarian follicles exert fluctuating tension in the tissue matrix and the dormant follicles in the ovary, transmitting pressure signals to the granulosa cells surrounding the oocytes, which are the main structures to absorb the mechanical stress. It has been shown that actin cytoskeleton serves as a pressure sensor to detect the environmental pressure and transmit the pressure signal intracellularly to alter the biological functions [53]. With abundant actin filaments in granulosa cells, it is likely that calponin 2 may regulate the actin cytoskeleton in sensing the pressure environment of ovarian follicles during folliculogenesis.

Three dimensional ultrasound studies showed that the volume of human ovary increases up to 70–80% of the original size before ovulation [54]. The expansion of the ovary is limited by the tunica albuginea and transfer the pressure back the interior of the ovary. The non-dominant ovarian follicles are the actual organs receiving the signals from intraovarian tension and be regulated. Actin microfilaments is the major cellular structure that stabilizes the delicate intracellular structure to maintain their functional integrity. A previous study found that actin polymerization-modulating drugs regulate ovarian follicle growth via the Hippo/YAP pathway [55]. Another study further demonstrated that modulating actin cytoskeleton dynamics induced disruption of Hippo pathway, leading to the production of cellular communication network (CCN) family proteins/connective tissue growth factors and anti-apoptotic baculoviral inhibitors of apoptosis repeat containing (BIRC) factor, which are factors activating the growth of secondary follicles [56].

Ovarian folliculogenesis is a highly dynamic process and needs intercellular communications between different types of the somatic cells as well as with the oocytes through the transzonal projections. The function of calponin 2 in non-muscle cells is based on its regulatory inhibition of actin-activated myosin motors. With important roles in bidirectional communication between oocytes and granulosa cells, transzonal projections with altered overall cytoskeletal tension due to the loss of calponin 2 may affect the interactions between oocytes and granulosa cells. In mammals, the primordial follicle pool is the origin of developing follicles and oocytes during the entire reproductive period. In an ovulation cycle, only limited number of the primordial follicles are activated and mature while most of primordial follicles remain in a quiescent state in the ovary. The exact mechanisms controlling the sequential release of primordial follicles are poorly understood. Recent studies proposed that intraovarian mechanical tension may play a role in maintaining most of the primordial follicles in resting state [9, 57].

With the literature evidence indicating the critical role of actin cytoskeleton in ovarian follicle activation and degeneration, the functions of calponin 2 in regulating mechanical tension in actin cytoskeleton [25, 26] support the hypothesis raised by the present study that calponin 2 plays a critical role in maintaining the structure and function of granulosa cells that are adapted to a physiologically fluctuating pressure environment. These findings provide a new direction for in depth mechanistic studies of POI.

## Methods

### Genetically modified mice

All animal procedures were performed using protocols approved by the Institutional Animal Care and Use Committee of Wayne State University. The mice were housed in a temperature and humidity-controlled room with a 12-hour light/dark cycle and free access to food and water. The generation of *Cnn2-*KO (*Cnn2* ^*−/−*^) mice has been described previously [28]. Briefly, two *loxP* sequences were inserted in tandem in intron 1 and intron 2 of the *Cnn2* gene for Cre recombinase-catalyzed deletion of exon 2. After transfection and cloning of embryonic stem cells, *Cnn2*-*flox* mice were generated in C57BL/6J strain. Disruption of *Cnn2* gene through deletion of the exon 2 segment was induced by crossing *Cnn2-flox* mice with oocyte-specific *Zp3-cre* mice (from Jackson Laboratory). Mice bearing the *Cnn2*-KO allele have been bred with C57BL/6J mice for > 15 generations to ensure uniform genetic background. The *Cnn2* ^+/−^ offspring were bred to obtain *Cnn2* ^−/−^ mice for phenotype studies. Genotyping of the mice used in the present study was done with tail biopsies from neonatal pups using PCR and reconfirmed in all experimental mice post-mortem by Western blot analysis of protein extracts from the spleen [36].

### Breeding studies

Reproductive performance of *Cnn2-*KO mice was examined in mating pairs of homozygous (*Cnn2* ^*−/−*^), heterozygous (*Cnn2* ^*+/−*\^) and/or wild type (WT, *Cnn2* ^+/+^) littermates. Homozygous *Cnn2-*KO male and female mice were paired to investigate the impact of calponin 2 deletion on fecundity. The male breeders used were less than 12 months old and proven fertile with viable offspring. Female mice reach sexual maturity at around 4 weeks old [58] and female breeders are usually retired from breeding at around 8–9 months old due to a sharp declining in reproductivity [59]. Therefore, 1–9 months old female mice were studied. Female breeders were checked for the presence of vaginal plug every morning between 8 and 10 am. The female breeders with confirmed vaginal plugs were placed in an individual cage. After term deliveries, all pups were genotyped and recorded for general health information.

To investigate if any effect of calponin 2 deletion on embryonic development, heterozygous *Cnn2-*KO male and heterozygous *Cnn2-*KO female mice were bred as above to examine whether the genotypes of the pups follow Mendelian distribution. To verify whether the male factor affects the reproductive outcomes, homozygous *Cnn2*-KO male mice were bred with young WT female mice and compared the outcome with that of WT control the pairs.

### Collection of ovaries for histology and immunohistochemistry studies

At designated age, *Cnn2-*KO and WT mice were euthanized by cervical dislocation under isoflurane anesthesia and the body weight was recorded. A midline abdominal incision was made, and the ovaries were identified at the end of the uterine horns close to the lower pole of the kidney. After careful trimming to remove the periovarian fat and the oviduct, the ovarian bursae was opened to excise the ovary. The ovary was fixed in 4% paraformaldehyde in phosphate-buffered saline (PBS, pH 7.4) overnight and proceeded for paraffin embedding using standard protocol.

For histology study, the sections were stained with either Mayer’s Hematoxylin and eosin Y (H&E, Electron Microscopy Scientific, 87019) or Periodic Acid-Schiff (PAS) staining using protocols provided by the manufacturers. For immunohistochemistry studies, the paraffin sections were deparaffinized, gradually rehydrated, and treated with 1% H_2_O_2_. After washed three times with PBS containing 0.05% Tween-20 (PBS-T) for five minutes each, the ovary sections were processed for antigen retrieval by microwave boiling for 15 minutes in 10 mM sodium citrate buffer, pH 6.0, containing 0.05% Tween 20. After cooling and washing with PBS-T, the sections were blocked with 3% bovine serum albumin (BSA) in PBS-T for 30 minutes and incubated with the following primary antibodies: Anti-VASA (Sigma, AB4330), anti-ZP-3 (ThermoFisher, 21279) and anti-follicular stimulating hormone receptor (FSHR) (Sigma, PA5-50963) at 4°C overnight. After washing with PBS-T three times five minutes each, the sections were incubated with horse radish peroxidase (HRP)-conjugated goat anti-rabbit IgG secondary antibody (Sigma, 12–348) in PBS-T containing 0.1% BSA at room temperature for one hour. Washed with PBS-T again, the slides were developed with 3.3’-Diaminobenzidine (DAB, Sigma, D12384) in dark for one minute. Nuclear counterstain was performed with hematoxylin. Negative control slides were processed the same procedures without the primary antibodies.

### Ovarian follicle counts

To obtain total ovarian follicle count, the entire paraffin-embedded ovary was serial sectioned at 8 μm thickness. Ovarian follicles were counted on every 6th of the sections. Total ovarian follicle counts were calculated as the sum of all counted sections multiplied by 6 [43]. Ovarian follicles without an oocyte were excluded to avoid duplications. Ovarian follicles were further categorized into primordial, primary, secondary and antral follicles under a light microscope using criteria established by Pederson and Peters [60]. Briefly, primordial follicles are defined by having an oocyte surrounded by a single layer of flattened granulosa cells. Primary follicles have the oocyte surrounded by a single layer of cuboid granulosa cells. When the oocyte are surrounding by more than two layers of the granulosa cells, they are categorized as secondary follicles. Antral follicles are secondary follicles with an acellular fluid filled antrum within the follicle. Atretic follicles are defined by having the oocytes displaying dark staining, distorted or loss of the intracellular contents with an empty zona pellucida shell. MOFs are defined by more than one oocyte residing in a single ovarian follicle with clearly defined surrounding granulosa cells.

### Primary culture of cumulus cells

WT C57BL/6J mice were intraperitoneally injected with 10 IU pregnant mare serum gonadotropin (PMSG, Sigma-Aldrich, G4877). 48 h later, the mice were euthanized to collect the ovaries under sterile condition. After trimming the periovarian tissue, the ovaries were placed in a petri dish containing prewarmed sterile PBS. Antral follicles were identified as bulging out vesicles on the ovarian surface under a dissection microscope. The follicles were punctured with a 25-G needle to release the antral stage cumulus-oocyte-complexes (COCs) from the ovary. After puncturing all the visible ovarian follicles, only the COCs with oocyte larger than 80 μm in diameter were collected, washed and filtered through a 40 μm cell strainer (Sigma, CLS431750) to remove fine tissue debris. The collected COCs were digested with 0.3 mg/mL hyaluronidase in DMEM medium to separate the oocyte from the surrounding cells. The cumulus cells were collected and cultured in DMEM/F12 medium supplemented with 10% fetal bovine serum, penicillin, streptomycin and glutamine in a humidified incubator at 37 °C in 5% CO_2_. The culture media were changed every three to four days until the cells reaching ~ 80% confluence.

### Isolation of ovulated COCs and preimplantation embryos

Adult WT female mice were intraperitoneally injected with 10 IU of PMSG followed by 10 IU of human chorionic gonadotropin (hCG, Sigma-Aldrich, C8554) 48 h later. The mice were euthanized 13 h after hCG administration to collect the ovulated COCs from the ampullae with a pair of sterile hypodermic needles under a dissection microscope. Another group of female WT mice were mated with fertile males after hCG injection. The female mice with proven presence of vaginal plugs were euthanized 50–52 h after the hCG injection. Under sterile conditions, the oviducts were isolated, put in a sterile culture dish and flushed with HTF media from the fimbrial end of the oviduct to collect the 4 cell stage embryos. The collected COCs and embryos were immediately washed three times with PBS to remove the media and fixed in 4% paraformaldehyde for immunofluorescent studies.

### SDS-PAGE and Western blotting analysis

Sodium dodecyl sulfate-polyacrylamide gel electrophoresis (SDS-PAGE) and Western blotting were performed as described previously [28]. After three washes with PBS, monolayer cells in culture dish were directly lyzed in SDS-gel sample buffer (50 mM Tris-HCl, pH 8.8, 2% SDS, 140 mM β-mercaptoethanol, 0.1% bromophenol blue, 10% glycerol). The cell lysates were collected in Eppendorf tubes and heated at 80 °C for 5 min, passed through a 25-G needle for more than 20 times to shear chromosomal DNA and reduce viscosity, and clarified by centrifugation in a microcentrifuge at 20,000 x g for 5 min. The extracted proteins were run on 12% SDS-gel with acrylamide to bis-acrylamide ratio of 29:1 in a Laemmli discontinuous buffer system. The resolved gels were stained with Coomassie Blue R-250 to reveal the protein bands.

Duplicate gels were blotted on nitrocellulose membranes using a Bio-Rad semidry electrical transfer apparatus for Western blot analysis. The blotted membranes were blocked with 1% BSA in Tris-buffered saline (TBS, 150 mM NaCl and 50 mM Tris-HCl, pH 7.5) at room temperature for 30 min and incubated with a rabbit anti-calponin 2 polyclonal antibody (RAH2) [17] in TBS containing 0.1% BSA at room temperature for one hour. After three washes with TBS containing 0.05% Tween-20 for 7 min each and three washes with TBS for 3 min each, the membranes were incubated with alkaline phosphatase-labeled anti-rabbit IgG secondary antibody (Sigma, A2306) at room temperature for one hour. Washed again as above, the membranes were developed in 5-bromo-4-chloro-3-indolyl phosphate/nitro blue tetrazolium chromogenic substrate solution to visualize the calponin 2 band [24].

### Immunofluorescence staining

Isolated mouse granulosa cells were cultured on sterile cover slips (Fisher Scientific) until reaching 50% confluency. The adherent cells were washed three times with PBS and fixed in 4% paraformaldehyde in PBS for 10 min. After washed three times five minutes each with PBS-T, the cells were incubated with 0.5% Triton X100 in PBS for 10 min and then, blocked with 3% BSA in PBS-T for 30 min. Washed again with PBS-T three times five minutes each, the slides were incubated with anti-calponin 2 monoclonal antibody 1D11 [24] in a humidified chamber at room temperature for one hour. After washing three times with PBS-T for 5 min each, the coverslips were incubated with fluorescein isothiocyanate-conjugated goat anti-mouse IgG (Sigma, F1010) or tetramethyl rhodamine isothiocyanate (TRITC)-conjugated anti-mouse IgG (Sigma, T-5393) in a dark box at room temperature for one hour. Rhodamine phalloidin (Life Technology, R37112) was used to stain the actin cytoskeleton. After final wash of three times 5 min each, the coverslips were mounted with ProLong Gold antifade reagent containing 4′,6-diamidino-2-phenylindole (DAPI) counter stain for nuclei (Molecular Probes by Life Technologies, ThermoFisher Scientific) and sealed using nail polisher. Images were obtained using a Leica SD600 spinning disc confocal microscope and processed using image analysis software (MetaMorph NX).

To handle isolated COCs and 4 cell embryos, sterile Pasteur pipettes were prepared by heating using a gas burner to pull into appropriate diameter for aspirating the COCs, oocytes or embryos with the tip of the glass pipette fire polished to avoid mechanical damage. The COCs or embryos were pooled and processed for immunofluorescent staining as above. Hoechst 33342 (ThermoFisher, H3570) was used for staining the nuclei. After completing the staining procedures, the COCs or embryos were loaded within a marked area on a Superfrost Plus slide with a PAP pen. The marked areas create a space for COCs and embryos to maintain their three-dimensional structure for imaging after a cover slip is sealed in place with nail polisher.

### Statistical analysis

IBM SPSS Statistics Version 27 was used for statistical analysis. All quantitative results are presented as means ± standard deviation (SD) Comparison of continuous variables between two groups were conducted by using Student’s *t*-test. Scattered correlation plots were analyzed by linear regression and Pearson’s correlation for statistical significance. *p* < 0.05 was considered statistically significant for comparisons.

## Data Availability

Original data are available upon request.

## Abbreviations

POI: Premature ovarian insufficiency
*Cnn2*-KO: Calponin 2 knockout
WT: Wild type
PGCs: Primordial germ cells
PCR: Polymerase chain reaction
BSA: Bovine serum albumin
HRP: Horse radish peroxidase
TBS: Tris-buffered saline
MOFs: Multi-oocyte follicles
FSHR: Follicle stimulating hormone receptor
COCs: Cumulus-oocyte-complexes
PBS: Phosphate-buffered saline
DAB: 3.3’-Diaminobenzidine
PMSG: Pregnant mare serum gonadotropin
HCG: Human chorionic gonadotropin
PAGE: Polyacrylamide gel electrophoresis
TRITC: Tetramethyl rhodamine isothiocyanate
DAPI: 4′,6-Diamidino-2-phenylindole
PAS: Periodic Acid-Schiff
SD: Standard deviation
